# Time to adjuvant chemotherapy after cytoreductive surgery and survival outcomes in epithelial ovarian cancer: a retrospective cohort study

**DOI:** 10.3389/fonc.2026.1856055

**Published:** 2026-08-07

**Authors:** Kshitij Jamdade, Marios Katsanevakis, Tobechi Njoku, Nazifa Tasnim, Ambreen Yaseen, Oluwafemi Ogundiran, Sahana Subbiah, Mamuna Akram, Carmen Gan, Jafaru Abu, Santhanam Sundar, Srinivasan Madhusudan, Ketan Gajjar

**Affiliations:** 1 Department of Gynaecological Oncology, Nottingham University Hospitals National Health Service (NHS) Trust, City Hospital, Nottingham, United Kingdom; 2 Department of Gynaecological Oncology, Lancashire Teaching Hospitals National Health Service (NHS) Foundation Trust, Preston, United Kingdom; 3 Department of Obstetrics and Gynaecology, Nottingham University Hospitals National Health Service (NHS) Trust, City Hospital, Nottingham, United Kingdom; 4 Department of Obstetrics and Gynaecology, United Lincolnshire Hospitals, Lincoln, United Kingdom; 5 Department of Clinical Oncology, Nottingham University Hospitals, Nottingham, United Kingdom; 6 Department of Medical Oncology, Nottingham University Hospitals, Nottingham, United Kingdom; 7 Naaz-Coker Ovarian Cancer Research Centre, Biodiscovery Institute, School of Medicine, University of Nottingham, University Park, Nottingham, United Kingdom

**Keywords:** advanced epithelial ovarian cancer, cytoreductive surgery, overall survival, progression-free survival, time to chemotherapy

## Abstract

**Objectives:**

To evaluate the impact of the interval between maximal-effort cytoreductive surgery and initiation of adjuvant chemotherapy on progression-free survival (PFS) and overall survival (OS) in women with advanced epithelial ovarian cancer.

**Design and methods:**

A retrospective cohort study was conducted including 145 patients who underwent primary or interval cytoreductive surgery followed by adjuvant chemotherapy at a tertiary gynaecological oncology centre between 2016 and 2021. Time to chemotherapy (TTC) was categorised as ≤6 versus >6 weeks and ≤8 versus >8 weeks. Survival outcomes were assessed using Kaplan–Meier analysis and multivariable Cox proportional hazards regression.

**Results:**

The median age was 60 years, and most patients had FIGO stage IIIC disease (77.2%) and high-grade serous carcinoma (78.0%). Twenty-three patients (15.9%) underwent primary debulking surgery and 122 (84.1%) underwent interval debulking surgery. Complete gross resection (R0) was independently associated with improved overall survival (HR 2.92, 95% CI 1.66–5.14; p<0.001) and progression-free survival (HR 2.16, 95% CI 1.33–3.52; p=0.002). Initiation of adjuvant chemotherapy more than eight weeks after surgery was independently associated with shorter progression-free survival (HR 1.65, 95% CI 1.12–2.45; p=0.013) but was not associated with overall survival. Baseline CA125 >1000 U/mL independently predicted poorer progression-free survival (HR 1.68, 95% CI 1.11–2.56; p=0.016). Mutation status and surgical approach (primary versus interval debulking surgery) were not independently associated with survival following multivariable adjustment.

**Conclusions:**

Delayed initiation of adjuvant chemotherapy beyond eight weeks following maximal-effort cytoreductive surgery was independently associated with poorer progression-free survival but not overall survival. Complete gross resection remained the strongest prognostic factor for both survival outcomes. These findings support optimisation of postoperative treatment pathways to minimise avoidable delays in chemotherapy while maintaining safe postoperative recovery. Prospective multicentre studies are warranted to validate these findings.

## Introduction

Ovarian cancer is the sixth most common gynaecological cancer and the eighth most lethal cancer worldwide (1, 2). It is largely due to its late-stage diagnosis and high recurrence rates despite aggressive treatment. Combined with platinum- and taxane-based chemotherapy, primary debulking surgery (PDS) is currently the cornerstone of treatment for these patients (3, 4). To achieve complete cytoreduction, surgical approaches have progressively evolved to include extensive upper abdominal procedures (5, 6). However, increasing surgical complexity has been associated with significant postoperative morbidity and mortality (7–9). Neoadjuvant chemotherapy (NACT) is generally offered to patients who are unsuitable for primary surgery because of older age, poor performance status, significant comorbidities, anticipated high surgical complexity, or a low likelihood of achieving complete cytoreduction (10, 11). Adjuvant chemotherapy (ACT) plays a crucial role in eradicating both visible and microscopic residual disease following cytoreductive surgery (12).

However, the optimal timing for initiating adjuvant chemotherapy after surgery remains a subject of considerable debate. Delays in commencing systemic treatment may allow residual microscopic disease to proliferate, potentially compromising the effectiveness of subsequent therapy.

Several retrospective studies (13–16) have suggested that prolonged time to chemotherapy (TTC) may be associated with poorer progression-free survival (PFS) and overall survival (OS). Evidence suggests that delayed initiation of adjuvant chemotherapy after interval debulking surgery is associated with inferior survival outcomes, with some studies recommending commencement of chemotherapy within five to six weeks after surgery (17). Conversely, prospective data have demonstrated no significant survival advantage for initiating chemotherapy within 42 days once important clinical factors, including age, disease stage, surgical characteristics, and insurance status, are considered (18). Another study suggested that the optimal TTC may lie between 23 and 43 days (approximately 3–6 weeks), while initiating chemotherapy very early (<23 days) did not improve PFS (19). These conflicting findings highlight the ongoing uncertainty regarding the optimal timing of postoperative chemotherapy, particularly in patients undergoing maximal-effort cytoreductive surgery aimed at achieving complete gross resection (R0). Furthermore, several clinicopathological factors—including residual disease status, surgical approach, tumour biology, postoperative recovery, and baseline disease burden—may influence survival outcomes independently of TTC.

Despite the growing body of literature, relatively few studies have evaluated the independent impact of TTC within contemporary cohorts undergoing maximal-effort cytoreductive surgery at high-volume tertiary centres. Clarifying this relationship is important for interpreting postoperative treatment pathways while recognising that survival outcomes are influenced by multiple established prognostic factors.

Accordingly, we performed a retrospective cohort study of patients undergoing maximal-effort cytoreductive surgery for epithelial ovarian, fallopian tube, or primary peritoneal cancer at Nottingham University Hospitals between 2016 and 2021. The primary objective of this study was to evaluate the association between time to adjuvant chemotherapy (TTC) and the two predefined survival outcomes: progression-free survival (PFS) and overall survival (OS). Secondary exploratory analyses evaluated the association between survival outcomes and established clinicopathological variables, including residual disease status, surgical approach (primary versus interval debulking surgery), baseline CA125 level, and BRCA mutation status. This study was not designed to determine the causes of delayed chemotherapy initiation or to evaluate healthcare service delivery or perioperative optimisation pathways; therefore, conclusions regarding these aspects should be interpreted with caution.

## Methods and design

We conducted a retrospective cohort study of patients who underwent maximal-effort cytoreductive surgery for epithelial ovarian, fallopian tube, or primary peritoneal cancer at Nottingham University Hospitals between January 2016 and December 2021. Eligible patients were identified from institutional surgical and oncology databases and electronic patient records. Inclusion criteria were histologically confirmed epithelial ovarian, fallopian tube, or primary peritoneal carcinoma and receipt of primary or interval cytoreductive surgery followed by adjuvant platinum-based chemotherapy within the study period. Patients undergoing secondary cytoreductive surgery for recurrent disease, those with non-epithelial ovarian tumours, uterine malignancies, or non-gynaecological primary cancers were excluded. Histological subtypes were classified according to the final histopathology report. Tumours reported as adenocarcinoma not otherwise specified (NOS) were reviewed and included only when confirmed to be of female genital tract origin. The sample size of 145 patients reflected all eligible consecutive cases treated during the study period.

The study was conducted in accordance with the principles of Good Clinical Practice and the Declaration of Helsinki. As this study utilised anonymised retrospective data collected as part of routine clinical care and was undertaken as a registered institutional clinical audit, formal Research Ethics Committee approval and individual patient consent were not required in accordance with Nottingham University Hospitals NHS Trust governance policies. The study was registered as a clinical audit entitled “Impact of the Time to Initiate Chemotherapy after Cytoreductive Surgery in Ovarian Cancer Patients on Survival Outcomes” (Audit Registration ID: 25-224C).

Maximal-effort cytoreductive surgery was defined as an aggressive surgical approach aimed at achieving complete gross resection (R0) whenever feasible and included procedures such as extensive peritonectomy, upper abdominal surgery, bowel resection, splenectomy, liver mobilisation or resection, diaphragmatic surgery, and other multivisceral procedures where required to achieve tumour clearance. Residual disease was classified intraoperatively as complete gross resection (R0) or macroscopic residual disease (non-R0).

Demographic and clinicopathological variables extracted from electronic patient records included age at diagnosis, body mass index (BMI), menopausal status, ECOG performance status, Charlson Comorbidity Index, serum CA125 concentration at diagnosis, primary tumour site (ovary, fallopian tube, or primary peritoneum), presence of ascites, histological subtype, tumour grade, FIGO stage, BRCA mutation status, residual disease status, perioperative outcomes, and time to adjuvant chemotherapy (TTC). Postoperative complications were defined as adverse events occurring within 30 days of surgery and were recorded according to contemporaneous clinical documentation; where available, complications were graded according to the Clavien–Dindo classification.

Patients were followed through electronic health records and oncology clinic documentation until death or the last recorded follow-up. Progression-free survival (PFS) was defined as the time (months) from cytoreductive surgery to the first documented disease progression, recurrence, or death from any cause, whichever occurred first. Overall survival (OS) was defined as the time (months) from cytoreductive surgery to death from any cause. Patients who remained alive or progression-free at the last clinical review were censored at their most recent follow-up.

Disease progression or recurrence was determined using radiological evidence of new or progressive disease, rising tumour markers in conjunction with clinical assessment, or histological confirmation where appropriate, in accordance with institutional follow-up protocols.

### Statistical analysis

Data analysis was performed using RStudio (version 2024.12.1; Posit PBC, Boston, MA, USA).

The primary study objective was to evaluate the association between time to adjuvant chemotherapy (TTC) and the two predefined survival outcomes: progression-free survival (PFS) and overall survival (OS). Secondary exploratory analyses evaluated established prognostic factors, including residual disease status, surgical approach, BRCA mutation status, and baseline CA125 level.

TTC was analysed using clinically relevant thresholds of ≤6 versus >6 weeks and ≤8 versus >8 weeks. These thresholds were selected *a priori* based on previously published literature evaluating postoperative chemotherapy timing in advanced ovarian cancer and reflected contemporary institutional practice during the study period (2016–2021), when chemotherapy initiation following maximal-effort cytoreductive surgery was individualised according to postoperative recovery, patient fitness, comorbidities, treatment readiness, and multidisciplinary scheduling.

Kaplan–Meier survival curves were constructed for OS and PFS, stratified by TTC, residual disease status, and BRCA mutation status. Median survival estimates with 95% confidence intervals (CIs) were calculated, and differences between groups were assessed using the log-rank test.

Cox proportional hazards regression models were used to evaluate associations between clinicopathological variables and survival outcomes. Missing covariate data were handled using multiple imputation with the mice package in R. Variables with a univariable p-value <0.20 or recognised clinical importance were entered into multivariable models. Final models were derived using backward stepwise selection based on the lowest Akaike Information Criterion (AIC). Hazard ratios (HRs), 95% confidence intervals, and p-values are reported.

Associations between categorical variables were assessed using the chi-square test or Fisher’s exact test where appropriate. Continuous variables were analysed using independent t-tests or Mann–Whitney U tests according to data distribution. All statistical tests were two-sided, and statistical significance was defined as p<0.05.

Because the specific clinical and logistical reasons contributing to delayed chemotherapy initiation (including postoperative recovery, patient preference, oncology scheduling, and institutional capacity) were not prospectively or uniformly recorded, formal analyses of determinants of TTC were not undertaken and are acknowledged as a study limitation.

## Results

A total of 145 patients with advanced epithelial ovarian cancer were included in the study. The median follow-up, estimated using the reverse Kaplan–Meier method, was 63 months (IQR 45–72). Baseline demographic and clinicopathological characteristics are summarised in **Table 1**. The cohort was predominantly composed of women with high-grade serous carcinoma (78.0%), FIGO stage IIIC disease (77.2%), and those undergoing interval debulking surgery (84.1%). During follow-up, 80 patients (55.2%) experienced disease recurrence.

**Table 1:** Baseline demographic and clinicopathological characteristics.

Characteristic	N = 145
Age at diagnosis, median (IQR), years	60 (53–69)
BMI, median (IQR), kg/m²	26.0 (22.0–31.7)
Charlson Comorbidity Index, median (IQR)	7 (7–8)
Menopausal status, n (%)	
• Postmenopausal	113 (77.9)
• Premenopausal	32 (22.1)
ECOG performance status, n (%)	
• 0	87 (60.0)
• 1–2	34 (23.4)
• Unknown	24 (16.6)
CA125 at diagnosis, median (IQR), U/mL	429 (115–1,625)
Missing CA125 data	8
Primary tumour site, n (%)	
• Ovary	129 (89.0)
• Primary peritoneum	11 (7.6)
• Ovary or peritoneum	4 (2.8)
• Fallopian tube	1 (0.7)
Histological subtype, n (%)	
• High-grade serous carcinoma	111 (78.0)
• Low-grade serous carcinoma	11 (7.7)
• Clear cell carcinoma	4 (2.8)
• Mucinous carcinoma	4 (2.8)
• Other epithelial histology	11 (7.58)
• Unknown	4 (2.8)
Tumour grade, n (%)	
• Grade 1	15 (11.0)
• Grade 2	4 (2.9)
• Grade 3	117 (86.0)
• Unknown	10
FIGO stage, n (%)	
• IIIA	2 (1.4)
• IIIB	3 (2.1)
• IIIC	112 (77.2)
• IV	28 (19.3)
Mutation status, n (%)	
• No mutation detected	91 (62.8)
• BRCA1/BRCA2/Other pathogenic mutation	54 (37.2)
Disease recurrence, n (%)	80 (55.2)
Timing of surgery, n (%)	
• Primary debulking surgery (PDS)	23 (15.9)
• Interval debulking surgery (IDS)	122 (84.1)

### Surgical procedures and perioperative outcomes

The extent of maximal-effort cytoreductive surgery is summarised in **Table 2**. Liver mobilisation was the most frequently performed upper abdominal procedure, undertaken in 133 patients (91.7%), followed by bowel resection with primary anastomosis and/or stoma formation in 66 (45.5%), splenectomy in 57 (39.3%), pancreatic nodule resection in 17 (11.7%), liver nodule resection in 16 (11.0%), and bladder wall nodule resection in 13 (9.0%). Peritonectomy was performed in the majority of patients, reflecting the extensive peritoneal tumour burden requiring maximal-effort cytoreductive surgery.

**Table 2:** Extent of cytoreductive surgery and perioperative outcomes.

Non-gynaecological organ involvement and surgical procedures	
Procedure	n (%)
Liver mobilisation	133 (91.7)
Bowel resection with primary anastomosis and/or stoma formation	66 (45.5)
Splenectomy	57 (39.3)
Pancreatic nodule resection	17 (11.7)
Liver nodule resection	16 (11.0)
Bladder wall nodule resection	13 (9.0)

Perioperative outcomes are presented in **Table 3**. The mean operative time was 428 minutes (SD =+/-117), mean estimated blood loss was 1,268 mL (SD =+/-851), mean postoperative length of hospital stay was 14.9 days (SD =+/-10.7), and the mean interval from cytoreductive surgery to initiation of adjuvant chemotherapy was 59.9 days (SD =+/-42.1).

**Table 3:** Perioperative outcomes.

Variable	Value (mean+/-SD)
Operative time (minutes)	428 +/- 117 (n=126)
Estimated blood loss (mL)	1,268 +/- 851 (n=122)
Length of hospital stay (days)	14.9 +/- 10.7 (n=144)
Time to adjuvant chemotherapy (days)	59.9 +/- 42.1 (n=145)

These findings demonstrate the complexity of the surgical procedures undertaken within this cohort.

### Overall survival

The results of the univariable and multivariable Cox proportional hazards analyses for overall survival are presented in **Table 4**.

**Table 4:** Univariable and Multivariable Cox proportional hazards regression analysis for overall survival.

Variable	Category	Univariable HR (95% CI)	p-value	Multivariable HR (95% CI)	p-value
FIGO stage	I–III	Reference	—	Reference	—
	IV	1.50 (0.88–2.56)	0.13	1.65 (0.95–2.87)	0.077
Type of cytoreductive surgery	IDS	Reference	—	Reference	—
	PDS	0.51 (0.28–0.93)	0.029	0.54 (0.29–1.00)	0.051
Residual disease	Complete gross resection (R0)	Reference	—	Reference	—
	Residual macroscopic disease	2.60 (1.50–4.50)	<0.001	2.92 (1.66–5.14)	<0.001
Mutation status	No mutation	Reference	—	Reference	—
	BRCA/other mutation	0.70 (0.44–1.11)	0.13	0.63 (0.40–1.00)	0.052
Menopausal status	Premenopausal	Reference	—	—	—
	Postmenopausal	1.07 (0.65–1.78)	0.80	—	—
CA125 at diagnosis	≤1000 U/mL	Reference	—	—	—
	>1000 U/mL	0.98 (0.63–1.54)	>0.9	—	—
Time to chemotherapy	≤8 weeks	Reference	—	—	—
	>8 weeks	1.03 (0.67–1.61)	0.90	—	—
Postoperative complications	No	Reference	—	—	—
	Yes	0.78 (0.50–1.20)	0.30	—	—

HR, hazard ratio; CI, confidence interval; PDS, primary debulking surgery; IDS, interval debulking surgery; R0, complete gross resection.

Complete gross resection (R0) was independently associated with improved overall survival. Patients with residual macroscopic disease (non-R0) had significantly poorer overall survival in both the univariable analysis (HR 2.60, 95% CI 1.50–4.50; p<0.001) and the multivariable analysis (HR 2.92, 95% CI 1.66–5.14; p<0.001).

Patients undergoing primary debulking surgery (PDS) demonstrated improved overall survival compared with those undergoing interval debulking surgery (IDS) in the univariable analysis (HR 0.51, 95% CI 0.28–0.93; p=0.029). However, this association was no longer statistically significant following multivariable adjustment (HR 0.54, 95% CI 0.29–1.00; p=0.051).

Mutation status, FIGO stage, menopausal status, serum CA125 concentration at diagnosis, time to chemotherapy (>8 weeks) and postoperative complications were not independently associated with overall survival.

### Progression-free survival

The results of the univariable and multivariable Cox proportional hazards analyses for progression-free survival are presented in **Table 5**.

**Table 5:** Univariable and multivariable Cox proportional hazards regression analysis for progression-free survival.

Variable	Category	Univariable HR (95% CI)	p-value	Multivariable HR (95% CI)	p-value
Residual disease	Complete gross resection (R0)	Reference	—	Reference	—
	Residual macroscopic disease	2.11 (1.31–3.43)	0.003	2.16 (1.33–3.52)	0.002
Time to chemotherapy	≤8 weeks	Reference	—	Reference	—
	>8 weeks	1.55 (1.05–2.28)	0.027	1.65 (1.12–2.45)	0.013
CA125 at diagnosis	≤1000 U/mL	Reference	—	Reference	—
	>1000 U/mL	1.58 (1.05–2.38)	0.027	1.68 (1.11–2.56)	0.016
Mutation status	No mutation	Reference	—	Reference	—
	BRCA/other mutation	0.79 (0.53–1.17)	0.20	0.67 (0.45–1.01)	0.056
Menopausal status	Premenopausal	Reference	—	—	—
	Postmenopausal	1.15 (0.73–1.79)	0.50	—	—
Performance status	ECOG 0	Reference	—	—	—
	ECOG 1–2	0.89 (0.53–1.49)	0.60	—	—
Histological subtype	LGSC	Reference	—	—	—
	HGSC	2.03 (0.84–4.88)	0.11	—	—
	Mucinous	1.29 (0.34–4.82)	0.70	—	—
	Clear cell	3.25 (0.88–12.0)	0.077	—	—
	Other epithelial	1.06 (0.20–5.62)	>0.9	—	—
FIGO stage	I–III	Reference	—	—	—
	IV	1.25 (0.78–1.99)	0.30	—	—
Type of cytoreductive surgery	IDS	Reference	—	—	—
	PDS	0.84 (0.51–1.41)	0.50	—	—
Postoperative complications	No	Reference	—	—	—
	Yes	1.39 (0.95–2.03)	0.085	—	—

HR, hazard ratio; CI, confidence interval; PDS, primary debulking surgery; IDS, interval debulking surgery; R0, complete gross resection.

Residual macroscopic disease (non-R0) remained independently associated with poorer progression-free survival in both the univariable analysis (HR 2.11, 95% CI 1.31–3.43; p=0.003) and multivariable analysis (HR 2.16, 95% CI 1.33–3.52; p=0.002).

Initiation of adjuvant chemotherapy more than eight weeks after cytoreductive surgery was independently associated with poorer progression-free survival (univariable HR 1.55, 95% CI 1.05–2.28; p=0.027; multivariable HR 1.65, 95% CI 1.12–2.45; p=0.013).

Similarly, serum CA125 concentrations >1000 U/mL remained independently associated with shorter progression-free survival (multivariable HR 1.68, 95% CI 1.11–2.56; p=0.016).

Mutation status was not independently associated with progression-free survival after multivariable adjustment. Likewise, menopausal status, ECOG performance status, FIGO stage, histological subtype, type of cytoreductive surgery (PDS versus IDS), and postoperative complications were not significantly associated with progression-free survival.

### Kaplan–Meier survival analyses

During a median follow-up of 63 months (IQR 45–72), the median progression-free survival for the entire cohort was 24 months, while the median overall survival was 64 months, as estimated by Kaplan–Meier analysis.

**Figure 1:**
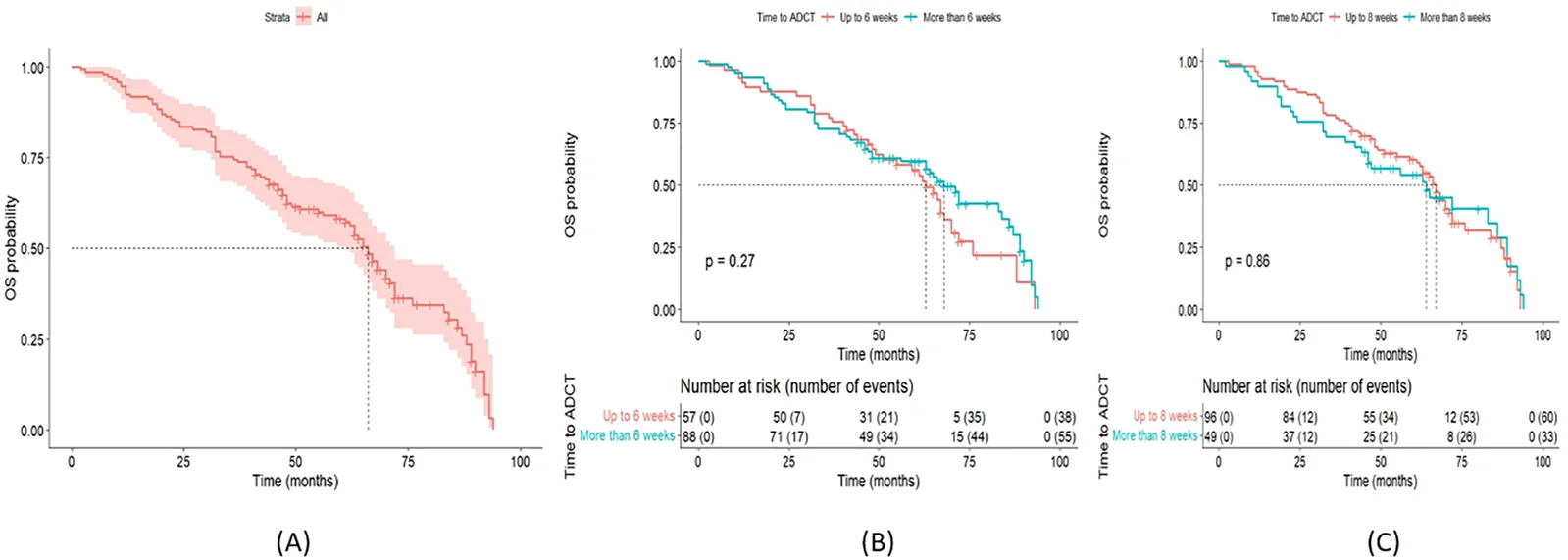
Kaplan–Meier overall survival analyses according to time to chemotherapy (TTC). **(A)** Kaplan–Meier curve demonstrating overall survival (OS) for the entire study cohort. **(B)** Kaplan–Meier comparison of overall survival according to time to initiation of adjuvant chemotherapy (TTC) ≤6 weeks versus >6 weeks following cytoreductive surgery. **(C)** Kaplan–Meier comparison of overall survival according to time to initiation of adjuvant chemotherapy (TTC) ≤8 weeks versus >8 weeks following cytoreductive surgery.

Kaplan–Meier survival curves demonstrating overall survival according to time to chemotherapy are shown in **Figures 1A, B**. No statistically significant difference in overall survival was observed between patients commencing adjuvant chemotherapy within or beyond six weeks (**Figure 1A**; log-rank p=0.27) or within or beyond eight weeks (**Figure 1B**; log-rank p=0.86) following cytoreductive surgery.

**Figure 2:**
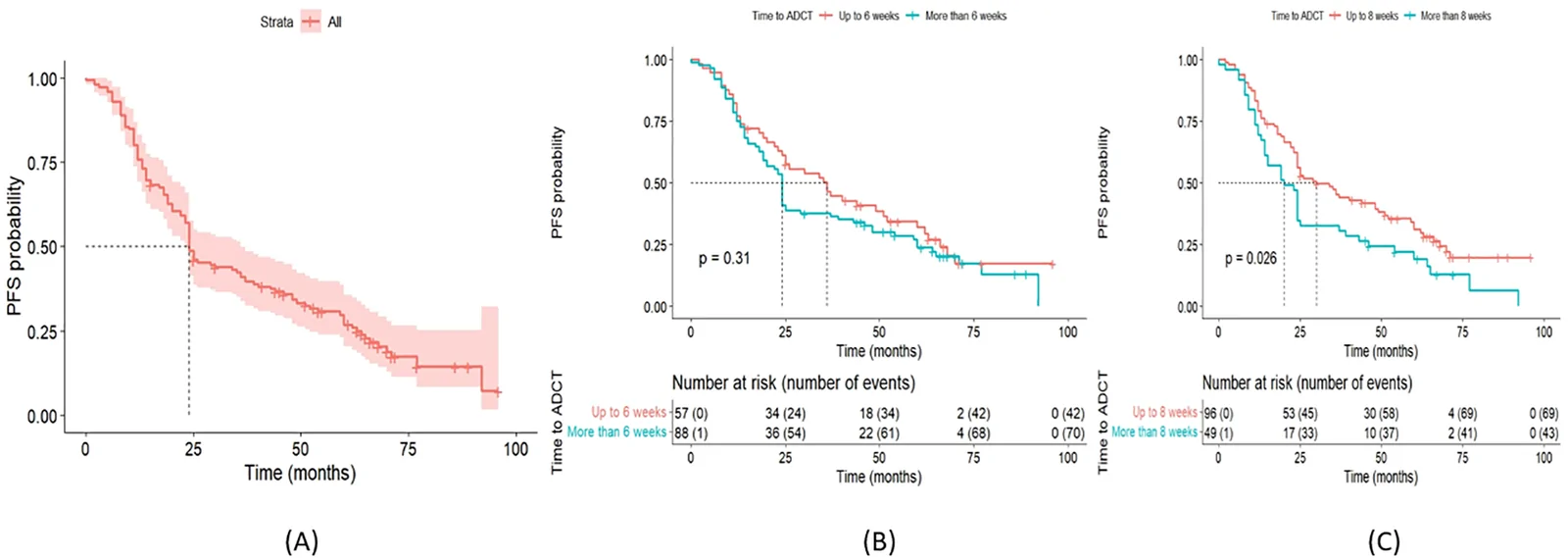
Kaplan–Meier progression-free survival analyses according to time to chemotherapy (TTC). **(A)** Kaplan–Meier curve demonstrating progression-free survival (PFS) for the entire study cohort. **(B)** Kaplan–Meier comparison of progression-free survival according to time to initiation of adjuvant chemotherapy (TTC) ≤6 weeks versus >6 weeks following cytoreductive surgery. **(C)** Kaplan–Meier comparison of progression-free survival according to time to initiation of adjuvant chemotherapy (TTC) ≤8 weeks versus >8 weeks following cytoreductive surgery.

Progression-free survival according to time to chemotherapy is illustrated in **Figures 2A, B**. No statistically significant difference in progression-free survival was observed using the six-week threshold (**Figure 2A**; log-rank p=0.31). However, patients commencing chemotherapy more than eight weeks after surgery demonstrated significantly shorter progression-free survival than those treated within eight weeks (**Figure 2B**; log-rank p=0.026).

**Figure 3:**
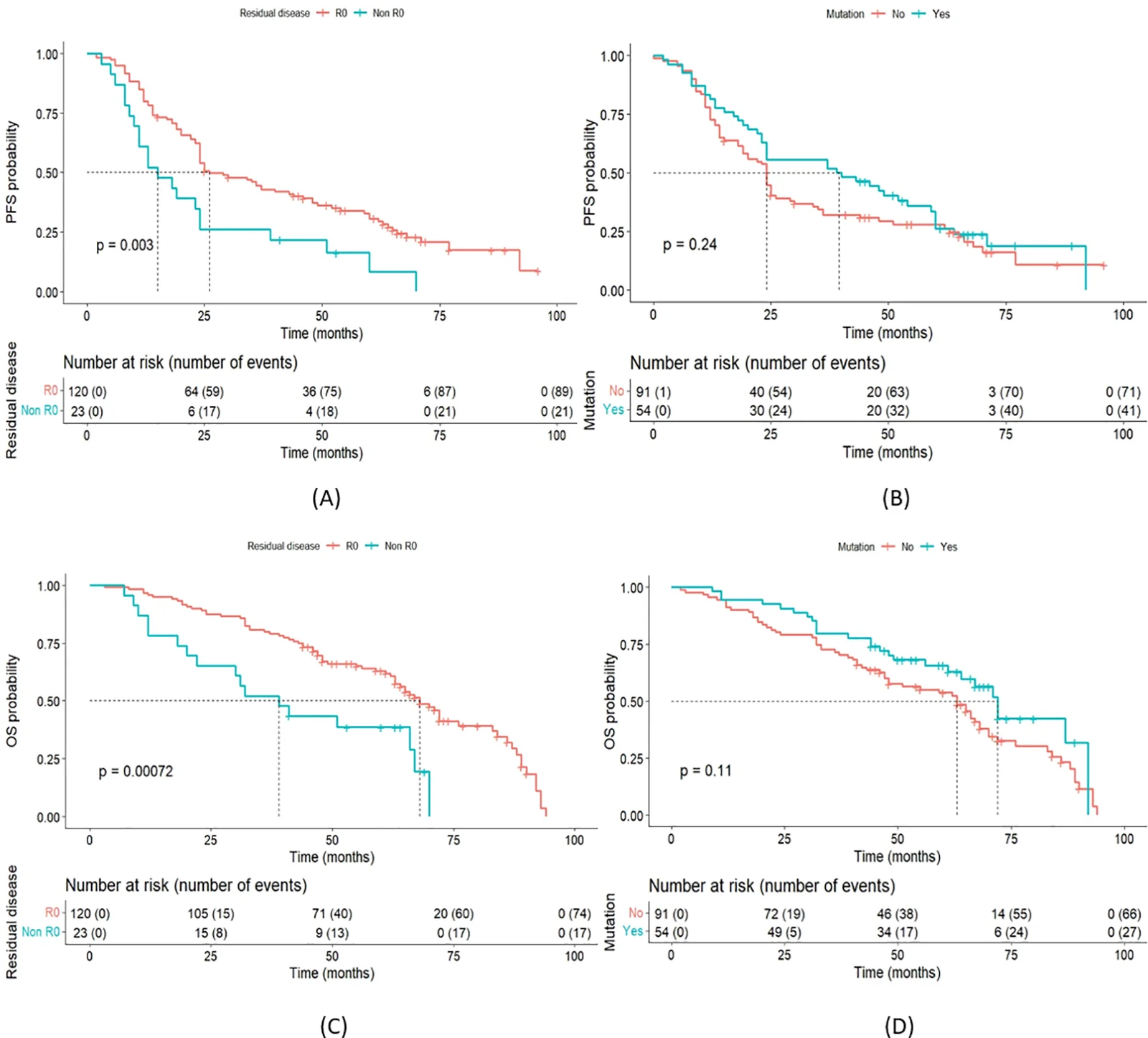
Kaplan–Meier survival analyses according to residual disease status and BRCA mutation status. **(A)** Kaplan–Meier comparison of overall survival according to residual disease status (R0 versus non-R0 resection) following cytoreductive surgery. **(B)** Kaplan–Meier comparison of overall survival according to mutation status (mutation detected versus no mutation detected). **(C)** Kaplan–Meier comparison of progression-free survival according to residual disease status (R0 versus non-R0 resection) following cytoreductive surgery. **(D)** Kaplan–Meier comparison of progression-free survival according to mutation status (mutation detected versus no mutation detected).

Kaplan–Meier analyses also demonstrated significantly improved progression-free survival (**Figure 3A**; log-rank p=0.003) and overall survival (**Figure 3C**; log-rank p=0.00072) among patients achieving complete gross resection (R0) compared with those with residual macroscopic disease. No statistically significant differences in progression-free survival (**Figure 3B**; log-rank p=0.24) or overall survival (**Figure 3D**; log-rank p=0.11) were observed according to mutation status.

## Discussion

The primary objective of this study was to evaluate the impact of time to initiation of adjuvant chemotherapy (TTC) following maximal-effort cytoreductive surgery on progression-free survival (PFS) and overall survival (OS) in women with advanced epithelial ovarian cancer. Our findings demonstrate that initiation of adjuvant chemotherapy beyond eight weeks was associated with shorter PFS after multivariable adjustment but not OS. In addition, complete gross resection (R0) remained the strongest independent prognostic factor for both PFS and OS, while elevated baseline CA125 (>1000 U/mL) was independently associated with inferior PFS. These findings reinforce the importance of achieving complete cytoreduction and minimising unnecessary delays in postoperative chemotherapy while recognising that survival outcomes are influenced by multiple tumour- and treatment-related factors.

The absence of a statistically significant association between TTC and OS suggests that delayed chemotherapy may predominantly influence the timing of disease recurrence rather than overall mortality. This observation may reflect the impact of subsequent lines of systemic therapy, maintenance treatments, patient selection, and post-recurrence management, all of which can modify long-term survival despite earlier disease progression.

Our findings are broadly consistent with previous studies evaluating the impact of TTC following cytoreductive surgery for advanced ovarian cancer, although the published evidence remains heterogeneous. Several retrospective studies have reported inferior survival among patients experiencing prolonged postoperative delays before commencing adjuvant chemotherapy, while others have demonstrated little or no association after adjustment for established prognostic factors.

In the present study, chemotherapy initiation beyond eight weeks was associated with shorter PFS after multivariable adjustment, whereas no statistically significant effect on OS was observed. These findings are consistent with reports by Aletti et al. (20), Gadducci et al. (21), Timmermans et al. (14) and Wright et al. (22), all of whom demonstrated poorer oncological outcomes with increasing delays in postoperative chemotherapy. In contrast, the Danish population-based study by Lydiksen et al. (23) reported no significant increase in mortality among patients commencing chemotherapy beyond 32 days, while Seagle et al. (24) observed an increased risk of death with delays exceeding 35 days but also suggested that initiating chemotherapy very early (<21 days) may adversely affect outcomes, possibly owing to incomplete postoperative recovery. Together, these studies suggest that both excessive delay and premature initiation of chemotherapy may be detrimental, highlighting the importance of balancing adequate postoperative recovery with timely systemic treatment.

The Gynaecologic Cancer InterGroup (GCIG) recognises progression-free survival as an appropriate endpoint for first-line ovarian cancer trials while acknowledging overall survival as the definitive measure of treatment efficacy (25). Our findings support this concept, demonstrating that TTC appears to have a greater influence on disease progression than on overall survival. This may reflect the increasing availability of effective maintenance therapies, subsequent platinum-based treatment, PARP inhibitors and other systemic therapies, which may attenuate differences in long-term survival following recurrence.

The selection of an eight-week threshold was based on previous published literature evaluating postoperative chemotherapy timing in advanced ovarian cancer, where delays beyond six to eight weeks have consistently been associated with inferior oncological outcomes. Although earlier thresholds (e.g. four weeks) have also been investigated, the majority of patients within our cohort commenced chemotherapy between six and eight weeks following surgery, making the eight-week threshold clinically relevant within our institutional practice. Nevertheless, we acknowledge that there is currently no universally accepted TTC threshold, and different cut-offs have been used across published studies, limiting direct comparison between cohorts.

The observed mean TTC of 59.9 days was longer than reported in several contemporary series. This finding should be interpreted in the context of the complexity of surgery performed within our cohort. Patients underwent maximal-effort cytoreductive procedures with a mean operative time of 428.1 ± 117.1 minutes and a mean estimated blood loss of 1267.6 ± 851.0 mL, reflecting the extensive nature of surgery undertaken. In addition, upper abdominal procedures, including liver mobilisation, were performed in a substantial proportion of patients (**Table 2**), further illustrating the complexity of surgical management within this tertiary referral centre. Because reasons for delay were not prospectively recorded, we cannot determine whether the prolonged TTC reflected postoperative recovery, institutional scheduling, referral pathways or patient-related factors. Furthermore, the specific clinical and logistical reasons contributing to delayed chemotherapy initiation, including postoperative recovery, patient preference, oncology scheduling and institutional capacity, were not prospectively or uniformly recorded. Consequently, formal analyses of determinants of TTC were not feasible, and the relative contribution of these factors to treatment delay could not be determined. Importantly, postoperative complications were not significantly associated with delayed chemotherapy initiation in our cohort, suggesting that factors beyond surgical morbidity may also influence TTC.

Complete gross resection (R0) remained the strongest independent prognostic factor for both progression-free and overall survival in our cohort. Patients with residual macroscopic disease experienced an almost threefold increased risk of death and more than a twofold increased risk of disease progression, emphasising that the quality of cytoreduction remains the single most important determinant of long-term oncological outcomes. These findings are consistent with extensive published evidence demonstrating that complete gross resection is the most powerful surgically modifiable prognostic factor in advanced epithelial ovarian cancer and remains the principal objective of maximal-effort cytoreductive surgery (26–29).

The observed association between delayed chemotherapy and poorer progression-free survival should therefore be interpreted within the context of surgical outcome. While timely initiation of chemotherapy is important (14, 15, 18, 20–24, 28), our findings suggest that achieving complete cytoreduction should not be compromised solely to facilitate earlier postoperative treatment. Rather, these findings support a treatment strategy that prioritises maximal surgical effort while minimising avoidable delays during postoperative recovery.

Primary debulking surgery demonstrated improved overall survival in the univariable analysis; however, this association was no longer statistically significant following multivariable adjustment. This finding should be interpreted cautiously. The comparison between PDS and IDS is inherently influenced by patient selection, disease distribution and tumour biology. Patients undergoing IDS generally presented with more extensive disease requiring neoadjuvant chemotherapy prior to surgery, whereas those selected for PDS were considered suitable for immediate maximal-effort cytoreduction. Furthermore, only 23 patients underwent PDS compared with 122 patients treated with IDS, substantially limiting statistical power and reducing the reliability of direct comparisons between surgical approaches. Consequently, our findings should not be interpreted as evidence favouring one surgical strategy over the other but rather reflect differences in patient selection within contemporary multidisciplinary ovarian cancer practice.

Baseline CA125 concentration greater than 1000 U/mL remained independently associated with poorer progression-free survival. This observation supports previous reports demonstrating that markedly elevated CA125 reflects greater tumour burden and more aggressive disease biology (30). Although CA125 is not sufficiently specific to guide treatment decisions independently, it continues to represent an inexpensive and widely available biomarker for prognostic stratification when interpreted alongside clinical, radiological and pathological findings.

Patients harbouring BRCA1, BRCA2 or other pathogenic mutations demonstrated numerically improved progression-free and overall survival; however, these associations did not reach statistical significance following multivariable adjustment. These findings should therefore be interpreted cautiously. The observed direction of effect is consistent with the well-recognised increased platinum sensitivity and improved outcomes reported among BRCA-mutated ovarian cancers (31, 32). However, mutation status was analysed collectively because of limited sample size, and homologous recombination deficiency (HRD) status was unavailable for most patients treated during the earlier years of the study period. Consequently, the prognostic contribution of individual molecular subgroups could not be explored within the present study.

The lack of a statistically significant association between mutation status and survival should therefore not be interpreted as evidence that molecular subtype lacks prognostic importance. Rather, it most likely reflects limited statistical power together with evolving molecular testing practices throughout the study period (2016–2021). As molecular profiling and maintenance therapies continue to evolve, future studies incorporating comprehensive genomic characterisation, including HRD testing, will provide a more refined understanding of the interaction between tumour biology, chemotherapy timing and survival outcomes (33, 34).

Although our findings highlight the importance of timely initiation of adjuvant chemotherapy following maximal-effort cytoreductive surgery, this study was not designed to determine the causes of delayed chemotherapy initiation or to evaluate healthcare service delivery, perioperative optimisation pathways or institutional processes influencing treatment timing. Accordingly, conclusions regarding these aspects should be interpreted with caution and warrant further prospective investigation.

The relatively prolonged median overall survival of approximately 64 months observed in this cohort likely reflects the high rate of complete gross resection together with contemporary systemic therapies available during follow-up. Under these circumstances, differences in time to chemotherapy may have a greater influence on progression-free survival than overall survival, which is also affected by subsequent lines of treatment and maintenance therapies.

## Clinical implications

The findings of this study have several practical implications for the multidisciplinary management of advanced epithelial ovarian cancer. First, they reinforce that complete gross resection should remain the primary surgical objective whenever safely achievable, as the survival benefit associated with R0 resection substantially outweighs the potential adverse effect of modest delays in postoperative chemotherapy (26–28).

Second, our findings support the initiation of adjuvant chemotherapy within eight weeks following maximal-effort cytoreductive surgery whenever clinically feasible. Delays beyond this interval were associated with significantly shorter progression-free survival, suggesting that unnecessary postponement of systemic treatment should be avoided through optimisation of perioperative pathways, multidisciplinary coordination and timely postoperative assessment.

Importantly, our findings should not be interpreted as supporting chemotherapy initiation before adequate postoperative recovery has occurred. Instead, they advocate a balanced, patient-centred approach in which chemotherapy is commenced as early as safely possible without compromising surgical recovery or increasing treatment-related morbidity. This is particularly relevant for patients undergoing extensive upper abdominal procedures, bowel resection or other complex components of maximal-effort cytoreductive surgery, in whom recovery may reasonably require several weeks.

Although the mean time to chemotherapy in our cohort (59.9 days) was longer than that reported in some contemporary series, postoperative complications were not independently associated with delayed chemotherapy initiation. This suggests that institutional processes, referral pathways and multidisciplinary scheduling may also contribute to treatment delays and represent potentially modifiable targets for quality improvement. Future service evaluation should focus on optimising postoperative care pathways to facilitate timely initiation of adjuvant chemotherapy while minimising avoidable treatment delays where clinically appropriate.

These findings highlight the importance of optimising postoperative treatment pathways and multidisciplinary coordination to facilitate timely initiation of adjuvant chemotherapy while avoiding unnecessary delays where clinically appropriate. However, this study was not designed to evaluate healthcare service delivery or institutional care pathways, and further prospective studies are required before conclusions regarding these aspects can be drawn.

## Study strengths

This study has several important strengths. It addresses a clinically relevant question regarding the optimal timing of adjuvant chemotherapy following maximal-effort cytoreductive surgery in women with advanced epithelial ovarian cancer, an area where published evidence remains inconsistent. The study cohort represents real-world clinical practice at a high-volume tertiary gynaecological oncology centre with extensive experience in maximal-effort cytoreductive surgery, thereby enhancing the clinical applicability of the findings.

A further strength is the comprehensive evaluation of multiple clinicopathological and treatment-related variables, including residual disease status, surgical approach, mutation status, perioperative outcomes and time to chemotherapy. The use of Kaplan–Meier survival analyses together with multivariable Cox proportional hazards modelling allowed adjustment for several recognised prognostic factors, improving the robustness of the survival analyses. Furthermore, multiple imputation was used to minimise bias arising from missing covariate data, strengthening the validity of the statistical analyses.

Importantly, our findings reinforce the established prognostic importance of complete gross resection while providing contemporary real-world evidence regarding the relationship between postoperative chemotherapy timing and oncological outcomes in patients undergoing maximal-effort cytoreductive surgery.

## Limitations

Several limitations should be considered when interpreting these findings. The retrospective, single-centre observational design precludes causal inference between time to chemotherapy (TTC) and survival outcomes. Although multivariable Cox regression was performed to adjust for recognised prognostic variables, residual confounding cannot be excluded. Unmeasured factors such as frailty, nutritional status, socioeconomic circumstances, postoperative functional recovery, institutional scheduling, referral pathways and access to subsequent therapies may all have influenced both chemotherapy timing and survival outcomes. In addition, confounding by indication remains an important consideration, as patients commencing chemotherapy later may have undergone more extensive surgery, experienced slower postoperative recovery or required additional optimisation before systemic treatment. Although postoperative complications were not significantly associated with delayed chemotherapy initiation in our cohort, other aspects of postoperative recovery were not captured and may have contributed to treatment delay.

The characteristics of our study cohort should also be considered when interpreting the findings. The majority of patients underwent interval debulking surgery following neoadjuvant chemotherapy, resulting in a marked imbalance between the primary debulking surgery (PDS) and interval debulking surgery (IDS) groups (23 versus 122 patients). While this reflects contemporary practice at our tertiary referral centre, it limits the statistical power of comparisons between surgical approaches and restricts the generalisability of conclusions regarding PDS versus IDS. Furthermore, the cohort included patients with primary peritoneal carcinoma and a small proportion of less common epithelial histological subtypes. Although these malignancies are managed using similar treatment principles, differences in tumour biology, chemosensitivity and prognosis may have introduced additional heterogeneity into the survival analyses.

The choice of the six- and eight-week TTC thresholds was based on previous published literature and institutional practice during the study period rather than biologically defined cut-off values. Earlier treatment intervals, such as four weeks, were not specifically evaluated, and different TTC thresholds have been adopted across published studies, making direct comparison between cohorts challenging. The mean interval from surgery to chemotherapy initiation in our cohort (59.9 days) was longer than that reported in several contemporary series. This likely reflects the complexity of maximal-effort cytoreductive surgery performed at our tertiary referral centre together with multidisciplinary postoperative assessment and institutional treatment pathways. However, the relative contributions of patient-related, surgical and healthcare system factors to delayed chemotherapy initiation could not be determined.

Molecular analyses were also limited by sample size and the availability of genomic data. BRCA1, BRCA2 and other pathogenic variants were analysed collectively because separate subgroup analyses were not feasible. Similarly, homologous recombination deficiency (HRD) status was unavailable for many patients treated during the earlier years of the study period. Consequently, the interaction between molecular subtype, chemotherapy timing and survival outcomes could not be explored in greater detail. Likewise, potentially informative secondary analyses—including interactions between TTC and residual disease status or BRCA mutation status, predictors of delayed chemotherapy initiation, and assessment of surgical complexity using the Aletti Surgical Complexity Score—could not be performed and should be considered in future prospective studies.

Finally, treatment pathways for advanced epithelial ovarian cancer evolved during the study period (2016–2021), including wider implementation of molecular testing, maintenance PARP inhibitors and changes in systemic treatment strategies. These developments may have influenced both progression-free and overall survival and could limit the applicability of our findings to contemporary practice. Nevertheless, this study provides contemporary real-world evidence from a high-volume tertiary referral centre, reinforcing the importance of complete gross resection and timely initiation of adjuvant chemotherapy while highlighting areas requiring further prospective investigation.

## Future research

Future prospective multicentre studies are needed to validate our findings and establish the optimal interval between maximal-effort cytoreductive surgery and initiation of adjuvant chemotherapy in women with advanced epithelial ovarian cancer (28, 29, 35, 36). Such studies should incorporate comprehensive assessment of surgical complexity, postoperative recovery, perioperative morbidity, patient frailty and healthcare system factors to better identify potentially modifiable causes of treatment delay.

Further research should explore whether the impact of time to chemotherapy differs according to residual disease status, molecular subtype, BRCA mutation status, homologous recombination deficiency (HRD) status and maintenance treatment strategies. Integration of contemporary molecular profiling, including HRD testing, together with evaluation of PARP inhibitor maintenance therapy and evolving systemic treatment approaches, may help identify patient subgroups most likely to benefit from expedited postoperative chemotherapy pathways and facilitate more individualised treatment planning.

Prospective studies evaluating the interaction between surgical quality, molecular characteristics and postoperative treatment pathways will be essential to optimise multidisciplinary care and improve long-term oncological outcomes in advanced epithelial ovarian cancer (29, 35, 36).

## Conclusion

In this retrospective cohort of women undergoing maximal-effort cytoreductive surgery for advanced epithelial ovarian cancer, initiation of adjuvant chemotherapy beyond eight weeks was independently associated with shorter progression-free survival but not overall survival. Complete gross resection remained the strongest independent prognostic factor for both survival outcomes. These findings support efforts to minimise avoidable postoperative delays in chemotherapy where clinically appropriate; however, prospective multicentre studies are required before specific time-to-chemotherapy thresholds can be recommended.

## Data Availability

The raw data supporting the conclusions of this article will be made available by the authors, without undue reservation.
