# Correction: Time to adjuvant chemotherapy after cytoreductive surgery and survival outcomes in epithelial ovarian cancer: a retrospective cohort study

**DOI:** 10.3389/fonc.2026.1972439

**Published:** 2026-09-03

**Authors:** Kshitij Jamdade, Marios Katsanevakis, Tobechi Njoku, Nazifa Tasnim, Ambreen Yaseen, Oluwafemi Ogundiran, Sahana Subbiah, Mamuna Akram, Carmen Gan, Jafaru Abu, Santhanam Sundar, Srinivasan Madhusudan, Ketan Gajjar

**Affiliations:** 1 Department of Gynaecological Oncology, Nottingham University Hospitals National Health Service (NHS) Trust, City Hospital, Nottingham, United Kingdom; 2 Department of Gynaecological Oncology, Lancashire Teaching Hospitals National Health Service (NHS) Foundation Trust, Preston, United Kingdom; 3 Department of Obstetrics and Gynaecology, Nottingham University Hospitals National Health Service (NHS) Trust, City Hospital, Nottingham, United Kingdom; 4 Department of Obstetrics and Gynaecology, United Lincolnshire Hospitals, Lincoln, United Kingdom; 5 Department of Clinical Oncology, Nottingham University Hospitals, Nottingham, United Kingdom; 6 Department of Medical Oncology, Nottingham University Hospitals, Nottingham, United Kingdom; 7 Naaz-Coker Ovarian Cancer Research Centre, Biodiscovery Institute, School of Medicine, University of Nottingham, University Park, Nottingham, United Kingdom

**Keywords:** advanced epithelial ovarian cancer, cytoreductive surgery, overall survival, progression-free survival, time to chemotherapy

**Figure 1** was incorrectly positioned in the “**Methods and design**” section of the published article. It should be relocated to the “*Kaplan–Meier survival analyses*” subsection of the **Results** section, alongside **Figures 2** and **3**.

In the **Methods and design** section, first paragraph, the last sentence - “The patient selection process is illustrated in **Figure 1**.” was intended to mention the Flow Diagram depicting the patient selection process, which has been omitted during the production process. This sentence should be removed.

A correction has been made to the section **Methods and design**, first paragraph:

“We conducted a retrospective cohort study of patients who underwent maximal-effort cytoreductive surgery for epithelial ovarian, fallopian tube, or primary peritoneal cancer at Nottingham University Hospitals between January 2016 and December 2021. Eligible patients were identified from institutional surgical and oncology databases and electronic patient records. Inclusion criteria were histologically confirmed epithelial ovarian, fallopian tube, or primary peritoneal carcinoma and receipt of primary or interval cytoreductive surgery followed by adjuvant platinum-based chemotherapy within the study period. Patients undergoing secondary cytoreductive surgery for recurrent disease, those with non-epithelial ovarian tumours, uterine malignancies, or non-gynaecological primary cancers were excluded. Histological subtypes were classified according to the final histopathology report. Tumours reported as adenocarcinoma not otherwise specified (NOS) were reviewed and included only when confirmed to be of female genital tract origin. The sample size of 145 patients reflected all eligible consecutive cases treated during the study period.”

The original version of this article has been updated.

